# Supplementary material for: Causal Relationship between Aging and Anorexia Nervosa: A White-Matter-Microstructure-Mediated Mendelian Randomization Analysis
Source: Biomedicines. 2024 Aug 16;12(8):1874. doi: 10.3390/biomedicines12081874 (PMC11351342; doi:10.3390/biomedicines12081874)
Supplement: Supplementary file 1 [file biomedicines-12-01874-s001.zip › Supplementary Figure/Figure S4/Singe SNP plot of MR analysis for TL to ubm-b-1781.pdf]

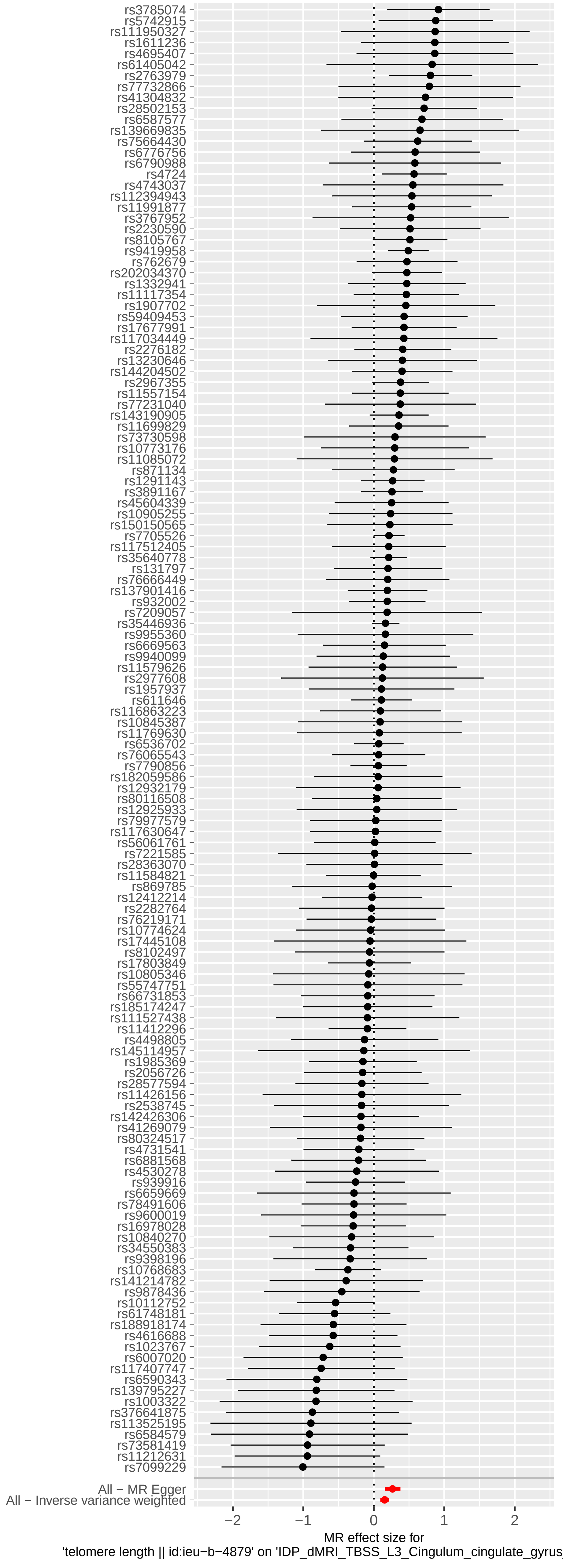
rs3785074
rs5742915
rs111950327
rs1611236
rs4695407
rs61405042
rs2763979
rs77732866
rs41304832
rs28502153
rs6587577
rs139669835
rs75664430
rs6776756
rs6790988
rs4724
rs4743037
rs112394943
rs11991877
rs3767952
rs2230590
rs8105767
rs9419958
rs762679
rs202034370
rs1332941
rs11117354
rs1907702
rs59409453
rs17677991
rs117034449
rs2276182
rs13230646
rs144204502
rs2967355
rs11557154
rs77231040
rs143190905
rs11699829
rs73730598
rs10773176
rs11085072
rs871134
rs1291143
rs3891167
rs45604339
rs10905255
rs150150565
rs7705526
rs117512405
rs35640778
rs131797
rs76666449
rs137901416
rs932002
rs7209057
rs35446936
rs9955360
rs6669563
rs9940099
rs11579626
rs2977608
rs1957937
rs611646
rs116863223
rs10845387
rs11769630
rs6536702
rs76065543
rs7790856
rs182059586
rs12932179
rs80116508
rs12925933
rs79977579
rs117630647
rs56061761
rs7221585
rs28363070
rs11584821
rs869785
rs12412214
rs2282764
rs76219171
rs10774624
rs17445108
rs8102497
rs17803849
rs10805346
rs55747751
rs66731853
rs185174247
rs111527438
rs11412296
rs4498805
rs145114957
rs1985369
rs2056726
rs28577594
rs11426156
rs2538745
rs142426306
rs41269079
rs80324517
rs4731541
rs6881568
rs4530278
rs939916
rs6659669
rs78491606
rs9600019
rs16978028
rs10840270
rs34550383
rs9398196
rs10768683
rs141214782
rs9878436
rs10112752
rs61748181
rs188918174
rs4616688
rs1023767
rs6007020
rs117407747
rs6590343
rs139795227
rs1003322
rs376641875
rs113525195
rs6584579
rs73581419
rs11212631
rs7099229
All − MR Egger
All − Inverse variance weighted
−2
−1
0
1
2
MR effect size for
'telomere length || id:ieu−b−4879' on 'IDP_dMRI_TBSS_L3_Cingulum_cingulate_gyrus_
